# Isolated Uhthoff phenomenon as first manifestation of multiple sclerosis: a case report

**DOI:** 10.1186/s12883-025-04193-6

**Published:** 2025-04-23

**Authors:** Annabelle Löffler, Charlotte Bereuter, Murat Delikaya, Judith Bellmann-Strobl, Frederike Cosima Oertel

**Affiliations:** 1 Experimental and Clinical Research Center, Max Delbrück Center for Molecular Medicine in the Helmholtz Association and Charité-Universitätsmedizin Berlin, Corporate Member of Freie Universität Berlin and Humboldt-Universität zu Berlin, Berlin, Germany; 2 Neuroscience Clinical Research Center, Charité-Universitätsmedizin Berlin, Corporate Member of Freie Universität Berlin and Humboldt-Universität zu Berlin, Berlin, Germany; 3 Department of Neurology, Charité-Universitätsmedizin Berlin, Corporate Member of Freie Universität Berlin and Humboldt-Universität zu Berlin, Berlin, Germany

**Keywords:** Uhthoff phenomenon, Multiple sclerosis, Optic neuritis, Case report

## Abstract

**Background:**

The Uhthoff phenomenon (UP) is a temporary worsening of neurological symptoms due to an increase in body temperature, commonly observed in demyelinating diseases, especially multiple sclerosis (MS).

**Case presentation:**

A 37-year-old female presented with a four-year history of transient vision impairment in the right eye, particularly during dancing. Visual evoked potentials (VEP) revealed prolonged P100 latency and reduced amplitude of the right eye. Optical coherence tomography (OCT) confirmed thinning of the macular ganglion cell and inner plexiform layer (GCIPL) and peripapillary retinal nerve fiber layer (pRNFL) in the right eye with significant inter-eye differences, consistent with a unilateral optic nerve lesion. Magnetic resonance imaging (MRI) findings of periventricular, pons and spinal cord lesions, along with positive oligoclonal bands in cerebrospinal fluid, confirmed MS diagnosis.

**Conclusions:**

This case underscores the importance of recognizing UP as a potential early indicator of MS. Specific diagnostic tools, such as OCT, VEP, and MRI, can be used to objectify optic nerve involvement and should be considered to identify underlying demyelinating conditions. Early diagnosis of MS can facilitate a timely initiation of therapies and improve patient outcomes. It is essential for clinicians to consider UP for better clinical management of MS.

## Background

The Uhthoff phenomenon (UP) describes a temporary worsening of neurological symptoms due to an increase in body temperature in demyelinating diseases, most frequently observed in multiple sclerosis (MS) [1]. MS is an inflammatory demyelinating disease of the central nervous system and one of the most common causes of neurological disability in young adults [2]. UP can occur due to fever, exercise, perimenstrual period, hot showers, sauna, sunbathing, infection, or other exposures to higher temperatures [3]. Besides the exacerbation of prior signs, new manifestations may also emerge. They frequently include visual disturbances such as blurred or double vision, fatigue, as well as cognitive symptoms like difficulty with memory and concentration, and motor symptoms such as weakness or difficulty with coordination and balance. It is reported that up to 80% of people with MS experience UP signs during their disease course, with many patients noting that even slight increases in body temperature can lead to significant symptom worsening [4]. Usually, the symptoms fully reverse once the body temperature returns to normal. Symptomatic treatment for UP is limited, and avoiding heat triggers interferes with the quality of life of affected patients by restricting their ability to engage in daily activities [5]. So far, the pathophysiological processes underlying UP remain unclear; temperature-dependent conduction block in demyelinated axons, along with associated changes in ion channel distribution and altered axonal conduction have been discussed as contributing mechanisms.

Herein, we report the case of a patient, who presented with transient visual disturbances of the right eye following physical activity over the past four years. The diagnosis of a right optic nerve lesion associated with UP was made based on optical coherence tomography (OCT), visual evoked potentials (VEP) and led together with findings from magnetic resonance imaging (MRI) and lumbar puncture to the MS diagnosis.

This case suggests that UP might be underrecognized by neurologists and ophthalmologists in clinical practice, when occurring isolated without other symptoms, delaying the referral for further diagnostic workup, such as neuro-visual assessments or MRI - and the start of treatment. This underscores the need for increased awareness among clinicians about the occurrence of this phenomenon preceding other MS signs for better diagnosis and disease management.

## Case presentation

A 37-year-old Caucasian female presented to our neuro-visual consultation with a four-year history of transient vision impairment in the right eye. She reported visual disturbances during summer heat and physical activity, particularly during dancing, which she pursued competitively. The symptoms included blurred vision across the entire field of vision, reduced contrast perception and increased glare. She had a high-demanding full-time employment but did not describe any association of visual disturbances with work-related stressors. The patient suffered from tension headaches, which she self-treated by applying heat to her neck. Neither the patient nor her family had any history of neurological or ophthalmological conditions. Prior diagnostic workup included doppler sonography of the extra- and intracranial vessels and neurological exams, which showed no pathological findings. An ophthalmological evaluation led to the prescription of glasses. Sarcoidosis was excluded only based on the clinical presentation and laboratory findings (Angiotensin-converting enzyme and soluble interleukin-2 receptor values within normal range). Additional diagnostic evaluation included testing for myelin-oligodendrocyte glycoprotein (MOG) antibodies and anti-aquaporin 4 antibodies, to rule out other demyelinating conditions, specifically MOG antibody-associated disease (MOGAD) and Neuromyelitis Optica Spectrum Disorders (NMOSD).

Through ocular examination we determined normal ocular motility without heterophoria or strabismus, normal anterior pole, and normal intraocular pressure (< 20 mmHg). High (HCVA) and low contrast visual acuity (LCVA) in both eyes were within the normal age range, with the right eye showing slightly lower acuity [HCVA [decimal]: 1.00; LCVA [decimal]: 0.63] than the left [HCVA [decimal]: 1.25; LCVA [decimal]: 0.80]. After physical effort (10 min moderate walk outside during warm spring weather), the visual acuity in the right eye was reduced [HCVA [decimal]: 0.80; LCVA [decimal]: 0.40]. The deterioration of visual function in heat could thus be objectified.

**Fig. 1:**
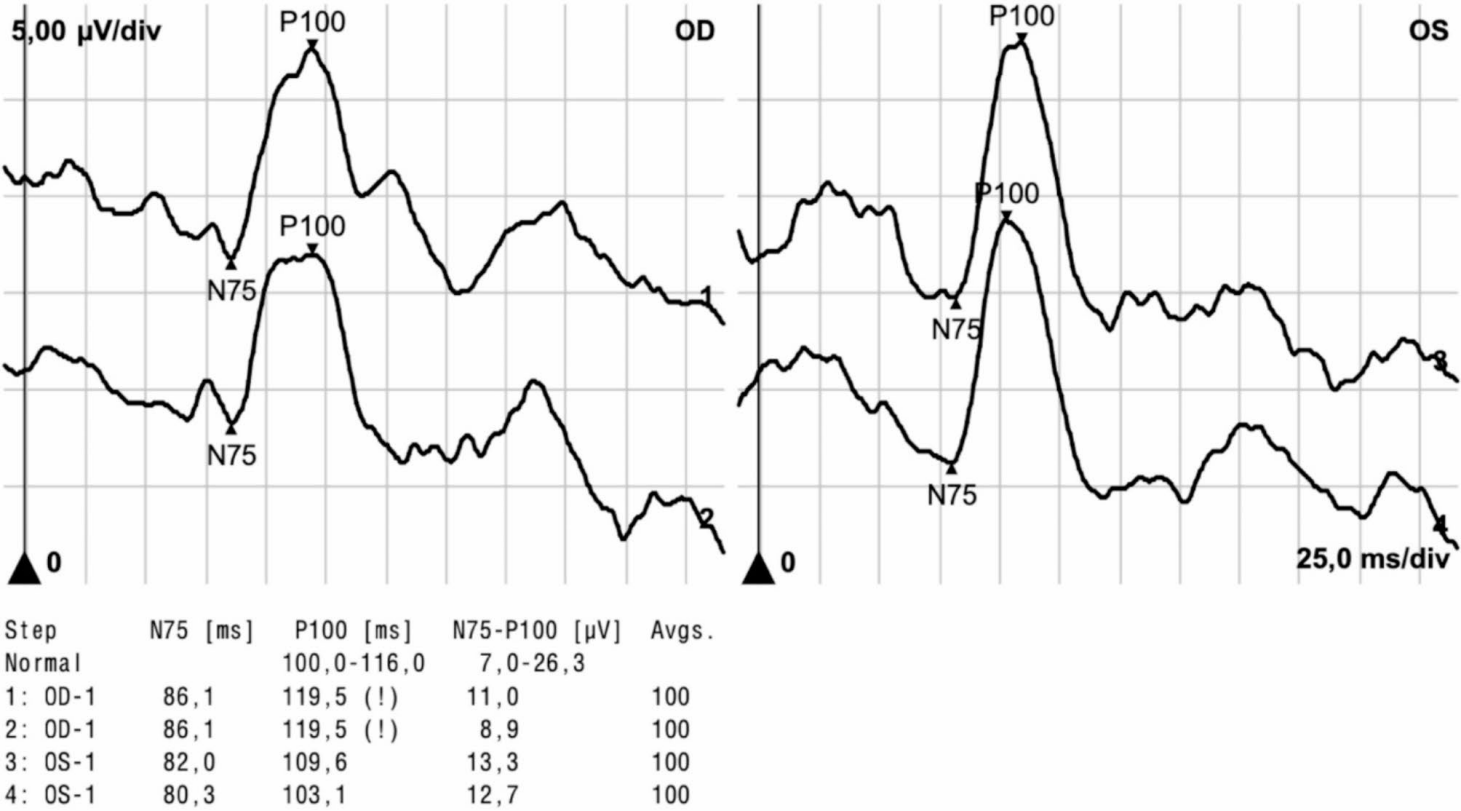
Pattern-reversal visual evoked potentials (VEP) measurements, for the right eye (OD) and the left eye (OS). The upper waveforms represent the first recordings and the lower waveforms the second recordings. The latencies are given in milliseconds (ms) for N75 and P100, and the amplitudes in microvolts (μV) for N75-P100.

Pattern-reversal VEP showed prolonged P100 latency (119.5 ms), exceeding the device’s threshold of 116.0 ms, and reduced amplitude (9.95 μV) on the right side compared to the left side (P100 latency: 106.35 ms, amplitude: 13 μV| inter-eye-difference: P100 latency: 13.15 ms, amplitude: 3.05 μV), indicating right optic nerve demyelination (see Fig. 1). We performed OCT (Spectralis SD-OCT, Heidelberg, Germany), which revealed unilateral macular and retinal nerve fiber layer (RNFL) thinning. Significant differences in RNFL and the combined ganglion cell and inner plexiform layer (GCIPL) thickness were observed between the two eyes. The 6 mm-diameter macular volume was reduced in the right eye (8.15mm^3^) compared to the left eye (8.34mm^3^). The average macular GCIPL thicknesses were 64.1 μm in the right eye and 69.9  μm in the left eye, with an absolute inter-eye difference of 5.8 μm.

**Fig. 2:**
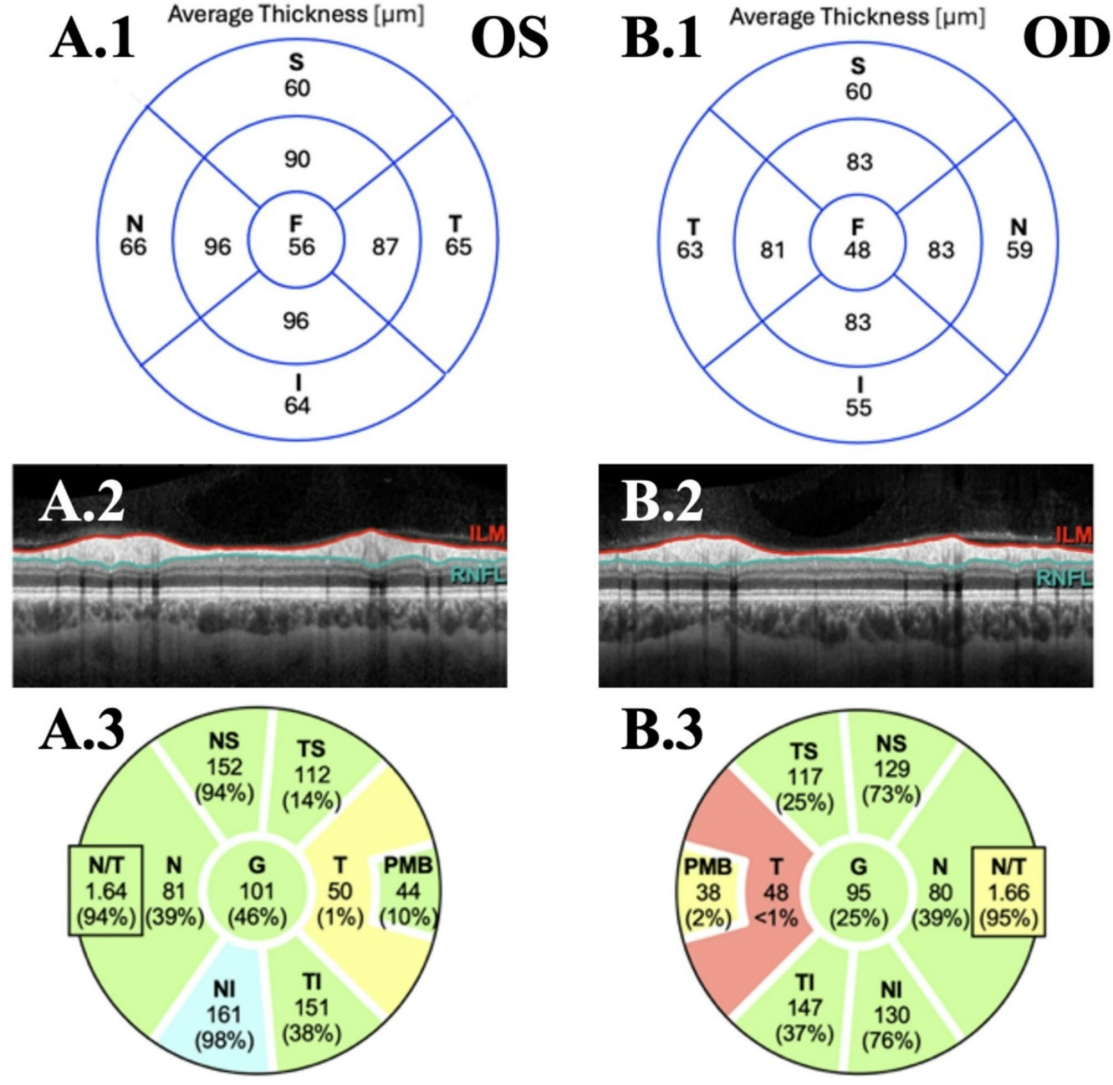
Macular and retinal OCT analysis of the patient, showing (**A**) the left eye and (**B**) the right eye. (1) Ganglion cell and inner plexiform layer thickness in μm for distinct sectors of the macula volume scan focused on the fovea (2) Representation of the peripapillary ring scan with marked boundaries of the retinal nerve fiber layer (RNFL). (3) Color-coded image of the RNFL thicknesses in comparison with normative device data: green: within normal range (> fifth percentile), yellow: borderline low (< fifth percentile), red: below normal range (< first percentile). Abbreviations: F foveal, S superior, I inferior, G global, NS nasal-superior, N nasal, NI nasal-inferior, TI temporal-inferior, T temporal, PMB papillomacular bundle, TS temporal-superior, RNFL retinal nerve fiber layer.

The average peripapillary RNFL (pRNFL) thickness measurements were 95 μm in the right eye and 101 μm in the left eye, with an absolute inter-eye difference of 6 μm. Significant thinning was especially observed in the temporal region and papillomacular bundle of the right eye (see Fig. 2). The unilateral VEP P100 latency prolongation, together with the inter-eye differences of GCIPL and pRNFL thicknesses indicate a previous lesion of the right optic nerve.

Subsequently, cranial and spinal MRI were performed. The cranial MRI showed a large periventricular lesion on the right parietal side along with smaller periventricular lesions. A small gliosis was noted in the right frontal white matter. Linear signal enhancement was observed in the pons on the right side. An asymmetrical signal alteration was found in the right retrobulbar optic nerve, with no pathological contrast enhancement along the nerve. Spinal MRI revealed two T2-hyperintense lesions, one at the level of T10 showed a left para-central signal enhancement, a second one at the level of T12 in the conus medullaris with round central signal enhancement. These findings validate both spatial and temporal dissemination, fulfilling the current criteria for MS [6]. Lumbar puncture was also performed indicating positive oligoclonal bands isolated in cerebrospinal fluid (CSF), further supporting the MS diagnosis.

In conclusion, the combination of clinical presentation, imaging (MRI, OCT), VEP and laboratory findings (CSF), revealed the diagnosis of MS with a right optic nerve lesion and UP as initial and isolated manifestation after a 4-year diagnostic delay. Supplementary diagnostic workup did not provide evidence of any alternative condition. The diagnosis was followed by detailed discussion of possible immunomodulatory treatment options.

## Discussion and conclusions

We describe the instructive case of a patient, where UP was the initial symptom of MS, and presented as isolated, transient, heat-induced unilateral visual disturbances without any other symptoms. Only a few similar cases of UP as the first manifestation of a demyelinating disease have been described in the literature. The incidence of UP remains unknown [7, 8]. For the first time we report a case of UP with significant diagnostic findings in OCT and VEP confirming an unilateral optic nerve lesion. In this case, the isolated presentation of UP led to a 4-year delay in diagnosis, underscoring how UP can easily be overseen by physicians, especially when it occurs prior to and without accompanying symptoms. This diagnostic delay postponed the start of therapy and risked disease progression and disability accrual. It is further atypical that despite MRI findings revealing contrast enhancement in the pons and spinal cord, there were no corresponding clinical deficits. This suggests that the lesion development in this patient is associated with mild functional loss, possibly indicating a form of MS with less destructive inflammation.

The use of specific diagnostic techniques such as OCT, MRI and VEP can identify prior optic nerve involvement and demyelinating lesions. The structural damage observed in MRI and OCT combined with the functional impairment demonstrated by VEP provided objective evidence for the diagnosis of MS. Recent revisions to the McDonald criteria for the diagnosis of MS may include the optic nerve as a fifth topography for demonstrating dissemination in space. In our case, the findings of significant inter-eye difference of pRNFL and GCIPL in OCT (with RNFL 6 μm and GCIPL > 4 μm), VEP P100 latency delay and optic nerve lesion identified on MRI, provided coherent evidence of a prior optic nerve lesion with all three modalities. These observations along with identified lesions in the infratentorial and periventricular region, fulfill spatial dissemination criteria as three out of at least two topographies are involved, confirming MS in accordance with the McDonald criteria 2017 [6] as well as with the proposed new criteria [9]. However, no clear clinical attack has been reported by this patient and UP is not explicitly mentioned as an attack presentation in the literature. Nonetheless in our case and as an extension of the 2017 diagnostic criteria, UP could be understood as an attack symptom rather than just being an indicative sign of prior ON, as it here represents an initial manifestation of MS and is supported by paraclinical findings. This case highlights how a complete study of the visual system with VEPs, OCT and MRI, as proposed in the new criteria, can be a key to diagnosing MS. The integration of the optic nerve, as another frequent location of demyelinating lesions, into the diagnostic criteria might support an earlier diagnosis and improve diagnostic performance, especially for patients presenting with ON or isolated visual symptoms [10]. This emphasizes the importance of visual pathway assessments to diagnose MS in cases like ours, where UP with visual disturbances was the first manifestation of the disease.

Several hypotheses, described in literature, attempt to explain the processes underlying UP– pathophysiologically often described as a change in or block of conduction. Demyelination could lead to alterations in ion channel distribution, such as the ectopic expression of voltage-gated potassium channels along axons, which may contribute to conduction failure, and is thought to worsen due to temperature sensitivity [3, 11]. Alternatively, temperature changes might induce direct alterations in axonal conduction, such as in axonal thresholds, refractory periods, and hyperpolarization. This could result in reduced excitability and impaired action potential propagation, potentially playing a role in the manifestation of UP [12]. Computational models also imply that temperature accelerates ion channel recovery processes beyond the rate of action potential initiation, disturbing axonal transmission and leading to propagation failure [13]. While multiple mechanisms have been suggested and might all partly contribute to the effect, the exact pathophysiological mechanisms underlying the UP are not fully understood and require further research.

This case report emphasizes the importance of recognizing UP as a potential early indicator of MS. Clinicians should employ specific diagnostic tools in case of suspected UP, including MRI, OCT, and VEP, to identify underlying demyelinating pathologies. The latter might also be employed before and after increasing the body temperature of patients employing light exercise. Early recognition of UP can facilitate MS diagnosis and timely initiation of treatment. Further research on the exact mechanisms underlying UP is still warranted.

## Data Availability

No datasets were generated or analysed during the current study.

## Abbreviations

CFS: Cerebrospinal fluid
CNS: Central nervous system
F: Foveal
G: Global
GCIPL: Combined ganglion cell and inner plexiform layer
HCVA: High contrast visual acuity
I: Inferior
LCVA: Low contrast visual acuity
MRI: Magnetic resonance imaging
MS: Multiple sclerosis
*N*: Nasal
NI: Nasal-inferior
NS: Nasal-superior
OCT: Optical coherence tomography
ON: Optic neuritis
PMB: Papillomacular bundle
pRNFL: Peripapillary retinal nerve fiber layer
S: Superior
T: Temporal
TI: Temporal-inferior
TS: Temporal-superior
UP: Uhthoff phenomenon
VEP: Visual evoked potential
